# Anti-aggressive effects of neuropeptide S independent of anxiolysis in male rats

**DOI:** 10.3389/fnbeh.2014.00185

**Published:** 2014-05-30

**Authors:** Daniela I. Beiderbeck, Michael Lukas, Inga D. Neumann

**Affiliations:** Department of Behavioral and Molecular Neurobiology, University of RegensburgRegensburg, Germany

**Keywords:** aggression, hypothalamus, nucleus accumbens, social behavior, anxiety, neuropeptide S

## Abstract

Neuropeptide S (NPS) exerts robust anxiolytic and memory enhancing effects, but only in a non-social context. In order to study whether NPS affects aggressive behavior we used Wistar rats bred for low (LAB) and high (HAB) levels of innate anxiety-related behavior, respectively, which were both described to display increased levels of aggression compared with Wistar rats not selectively bred for anxiety (NAB). Male LAB, HAB, and NAB rats were tested for aggressive behavior toward a male intruder rat within their home cage (10 min, resident-intruder [RI] test). Intracerebroventricular (icv) infusion of NPS (1 nmol) significantly reduced inter-male aggression in LAB rats, and tended to reduce aggression in HAB and NAB males. However, local infusion of NPS (0.2 or 0.1 nmol NPS) into either the nucleus accumbens or the lateral hypothalamus did not influence aggressive behavior. Social investigation in the RI test and general social motivation assessed in the social preference paradigm were not altered by icv NPS (1 nmol). The anti-aggressive effect of NPS is most likely not causally linked to its anxiolytic properties, as intraperitoneal administration of the anxiogenic drug pentylenetetrazole decreased aggression in LAB rats whereas the anxiolytic drug diazepam did not affect aggression in HAB rats. Thus, although NPS has so far only been shown to exert effects on non-social behaviors, our results are the first demonstration of anti-aggressive effects of NPS in male rats.

## Introduction

Aggressive behavior is an important pre-requisite for the acquisition and maintenance of feeding resources, territory and mating partners and, therefore, for the survival of an individual and the species. However, dysregulation of aggression among conspecifics can lead to severe injury and death. Neuropeptides, like arginine vasopressin and oxytocin, have been shown to be important regulators of inter-male, female and maternal aggression (Ferris, 2005; Neumann and Landgraf, 2012; Calcagnoli et al., 2013; De Jong et al., 2013), but are also part of neuronal circuits regulating anxiety in rats and mice (Landgraf et al., 1995; McCarthy et al., 1996; Bielsky et al., 2005; Blume et al., 2008). With respect to a possible link between inter-male aggression and anxiety, studies describe either an association between low levels of anxiety and high levels of aggression (Nyberg et al., 2003; Beiderbeck et al., 2007), or no such link (De Boer et al., 2003). In rats selectively bred for extremes in innate anxiety, low anxiety-related behavior (LAB) has been linked to high and abnormal inter-male aggression, but also male rats with high anxiety-related behavior (HAB) show higher aggression levels compared with rats not selectively bred for anxiety (NAB) (for review see Neumann et al., 2010). Co-selection of factors regulating aggression along with those regulating both, low and high, anxiety-related behavior is likely to underlie the behavioral phenotype of LAB and HAB rats.

The recently discovered neuropeptide S (NPS) exerts strong anxiolytic effects in rats, including LAB and HAB rats, and mice, when administered into the brain ventricles (icv), via the nasal route (Xu et al., 2004; Ionescu et al., 2012; Lukas and Neumann, 2012; Slattery et al., 2012) and locally into the amygdala and the hippocampus (Jüngling et al., 2008; Slattery et al., 2012; Dine et al., 2013). NPS acts via specific G protein-coupled receptors (NPSR) (Xu et al., 2004; Reinscheid et al., 2005), which are widely localized within limbic and hypothalamic brain regions, whereas NPS mRNA expression is restricted to a small population of neurons between the locus coeruleus and the Barrington nucleus (Xu et al., 2004; Leonard and Ring, 2011). In addition to its anxiolytic properties, NPS facilitates spatial learning and memory in the Morris water maze (Han et al., 2009), the extinction of aversive memories (Jüngling et al., 2008) as well as object recognition memory (Okamura et al., 2011; Lukas and Neumann, 2012). However, the anxiolytic and memory enhancing effect of NPS were exclusively seen in a non-social context, as neither social anxiety in the social preference/social avoidance paradigm nor social memory in the social discrimination test were altered by NPS (Lukas and Neumann, 2012).

Furthermore, NPS was shown to inhibit reward and addiction behaviors. Thus, NPS inhibits morphine-induced conditioned place preference in mice (Li et al., 2009) and reduces alcohol intake in an alcohol-preferring rat breeding line (Badia-Elder et al., 2008). In this context it is of interest that the high aggression of LAB rats is driven by an increased neuronal activity in the nucleus accumbens (NAc), part of the reward circuitry (Beiderbeck et al., 2012). High aggression in LAB rats was also accompanied by an increased neuronal activation in hypothalamic subregions including the anterior and lateral hypothalamus (LH) (Beiderbeck et al., 2012)—regions that are also activated after icv NPS administration (Kallupi et al., 2010) and express high levels of NPSR mRNA (Leonard and Ring, 2011). This makes NPS a promising candidate for reducing inter-male aggression in highly aggressive individuals.

The primary aim of this study was, therefore, to investigate the effect of NPS on inter-male aggression in LAB and HAB rats as models of hyper-aggression, with non-selected (NAB) rats serving as low-aggressive controls. Additionally, we aimed to localize the putative anti-aggressive effects of NPS in promising target regions that are important in the regulation of both reward and aggression, i.e., the NAc and LH. Finally, to investigate a putative link between aggression and anxiety, we monitored inter-male aggressive behavior after treatment with established anxiolytic or anxiogenic agents.

## Materials and methods

### Animals

Experiments were carried out on male Wistar rats either selectively bred for low (LAB) and high (HAB) anxiety-related behavior or non-selected rats (NAB) in the animal facilities of the University of Regensburg (Neumann et al., 2010). Rats were housed in groups of 4–6 under standard laboratory conditions (12:12 h light/dark cycle with lights on at 06:00 h, 21 ± 1°C, 60 ± 5% humidity, standard rat chow and water *ad libitum*). For behavioral testing, adult resident LAB, HAB, and NAB male rats (350–450 g) were each housed in an observation cage (40 × 24 × 35 cm) together with a female Wistar rat (Charles River, Sulzfeld, Germany) for 10 days (12:12 h light/dark cycle with lights off at 12:00) to stimulate territorial behavior (Flannelly and Lore, 1977; Beiderbeck et al., 2012). Bedding was not changed during the last 3 days prior to the resident-intruder (RI) test. All tests took place during the active phase starting 1 h after lights off, i.e., between 13:00 and 15:00 h. The experiments were approved by the Committee on Animal Health and Care of the Government of the Oberpfalz.

### Intracerebral implantation of guide cannulas

For intracerebral drug infusion, guide cannulas were stereotaxically implanted either unilaterally 2 mm above the lateral ventricle or bilaterally 2 mm above the NAc or the LH [relative to bregma; icv: 1.0 mm posterior, 1.6 mm lateral, 2.0 mm deep; NAc: 1.7 mm anterior, 1.6 mm lateral, 4.6 mm deep; LH: 1.8 mm posterior, 1.8 mm lateral, 6.0 mm deep; nose −3.5 mm, (Paxinos and Watson, 1998)]. Rats were anesthetized (Isoflurane, Forene®, Abbott GmbH and Co. KG, Wiesbaden, Germany), injected with an antibiotic (Baytril®, Bayer Vital GmbH, Leverkusen, Germany), and mounted on a stereotaxic frame. The guide cannula (for icv: 21 G, 12 mm; for NAc and LH: 23 G, 12 mm; Injecta GmbH, Germany) was fixed to the skull with two jeweler's screws and dental cement (Kallocryl, Speiko-Dr. Speier GmbH, Muenster, Germany) and closed by a stainless steel stylet (25 G and 27 G, respectively). Three days prior to and one day after surgery, rats were handled (stroking, holding, cleaning of stylets) for a total of 4 days to minimize non-specific stress responses during the experiment.

### Intracerebral drug application

In order to study the effects of intracerebral NPS infusion, either icv, or directly into the left and right NAc, or the left and right LH, on aggression and social approach behavior, rats received either synthetic NPS (icv: 1 nmol/5 μl, i.e., 2 μg/5 μl; NAc/LH: 0.1 or 0.2 nmol/1 μl, i.e., 0.2 or 0.1 μg/1 μl; H-6164; Bachem Holding AG, Bubendorf, Switzerland) or vehicle (VEH, sterile Ringer's solution, pH 7.4, B. Braun Melsungen, Germany) via an infusion cannula inserted into the guide cannula and connected to a Hamilton syringe via polyethylene tubing. After icv or local infusion, the cannula was left in place for 30 s. Infusions were performed 30 min prior to behavioral testing [RI test, elevated pluz-maze (EPM), social preference test]. Doses and time points for icv and local infusions were chosen based on their behavioral effects in an emotional (EPM) and learning (object recognition) context (Lukas and Neumann, 2012).

### Intraperitonal (ip) injections

The anxiogenic drug pentylenetetrazole (PTZ; 25 mg/kg; Sigma-Aldrich, Steinheim, Germany) and the anxiolytic drug diazepam (DIA; 2 mg/kg; Ratiopharm, Ulm, Germany) or VEH were injected ip 30 min prior to behavioral testing on the EPM or in the RI test. Based on Pellow et al. (1985) (20 mg PTZ/kg 5 min before EPM) we tested a dose of 25 mg/kg PTZ 30 min before EPM exposure in a pilot study in order to match the time point of our other experiments; convulsive effects were not found in any of the tested rats. To minimize unspecific stress responses due to the injection, rats were handled daily during the early dark phase starting 3 days prior to the experiment by gently placing the rat in a dark tube (one end closed; length: 17 cm; diameter: 8 cm) and touching their belly. As a result all rats went into the tube voluntarily without signs of arousal or resistance.

### Behavioral paradigms

#### Resident-intruder (RI) test

For behavioral testing of male rats without prior surgery, the female was removed from the resident's cage 30 min before the beginning of the RI test. Otherwise, the female rat was removed from the resident's cage during surgery, and males were single-housed in their experimental observation cage thereafter. At the start of the 10-min RI test, an unfamiliar, non-aggressive, lighter (10% less body weight) male Wistar rat (Charles River, Sulzfeld, Germany) was placed into the cage of the resident.

The behavior of the resident was videotaped, and the following behaviors were scored by an experienced observer blind to breeding line and treatment: aggressive behavior (attack, lateral threat, offensive upright, keep down, threat, mounting, aggressive grooming), social investigation (investigating opponent, anogenital sniffing), exploration, self-grooming, defensive behavior, immobility and other behaviors like food intake or digging (Koolhaas et al., 1980; Beiderbeck et al., 2012). Behavior was scored in real-time using pre-set computer keys (Eventlog; Version 1.0, 1986, R. Hendersen).

#### Social preference test

The effects of icv NPS on social approach/social avoidance behavior was studied using the social preference paradigm (Lukas et al., 2011). Briefly, rats were placed in a novel arena (40 × 80 × 40 cm, red light), and after 30 s of habituation, an empty wire-mesh cage (non-social stimulus; 20 × 9 × 9 cm) was placed at one side wall of the arena for 4 min. The empty cage was then exchanged by an identical cage containing an unknown adult male Wistar rat (social stimulus) for 4 min. Exploration times of the non-social and social stimulus (i.e., the time the rat spent in active olfactory investigation) were scored by an observer blind to the treatment using JWatcher behavioral observation software (V 1.0, Macquarie University and UCLA). Data are presented as the percentage of time investigating the non-social vs. the social stimulus, i.e., investigation time/total time (4 min) × 100. A significantly higher percentage of mean investigation of the social vs. the non-social stimulus within one group of rats was considered social preference. Before each trial, the arena was cleaned with water containing a low concentration of detergent.

#### Elevated plus-maze (EPM)

The effects of local (NAc, LH) NPS, ip PTZ and ip DIA on non-social anxiety-related behavior was assessed using the EPM (Pellow et al., 1985), which consisted of two opposing open (50 × 10 cm, 100 lux) and two opposing closed arms (50 × 10 × 40 cm, 20 lux) connected by a central area (Beiderbeck et al., 2007). Briefly, the EPM was made of dark gray plastics, elevated 80 cm above the floor, and surrounded by an opaque curtain to avoid external disturbance. Before each trial, the maze was cleaned with water containing a low concentration of a detergent. The rat was placed in the central area facing a closed arm. The percentage of time spent on the open arms during the 5-min test (time on open arms/time on open and in closed arms × 100) was assessed as anxiety-related behavior, and the number of entries in the closed arms as measurement for locomotion. Behavior was recorded by means of a video camera mounted above the platform and scored by a trained observer (Plus-maze version 2.0; E. Fricke).

### Experimental design

By performing an initial RI test before beginning of all the experiments, we confirmed that the level of aggression in both the future vehicle and treatment groups did not differ.

### Experiment 1: effects of icv NPS on inter-male aggression and general social motivation

In order to study the effects of icv NPS on territorial aggression, groups of male LAB (*n* = 20), HAB (*n* = 20), and NAB (*n* = 18) residents were infused icv with either NPS (1 nmol/5 μl) or VEH (Ringer' solution) 30 min prior to the RI test. To further test for the specificity of NPS effects on aggressive behavior, the general social motivation of several of these LAB (*n* = 19) and HAB (*n* = 16) rats was tested in the social preference test 30 min after icv infusion of either NPS or VEH two days after the RI test has been performed. Rats were infused with the identical icv treatment received prior to the RI test.

### Experiment 2: effects of infusion of NPS into the NAc and LH of LAB rats

In order to localize the anti-aggressive effects of NPS, either NPS (0.1 nmol/1 μl, 0.2 nmol/1 μl), or VEH were bilaterally infused into the NAc (*n* = 18) or the LH (*n* = 46) of aggressive LAB rats. Both brain regions were chosen based on high local NPSR expression (Leonard and Ring, 2011) and an increase in neuronal activation in response to the display of high aggression in LAB rats (Beiderbeck et al., 2012). Three days later, several of these rats (NAc, *n* = 18; LH, *n* = 22) were tested on the EPM for anxiety-related behavior.

### Experiment 3: effects of anxiogenic (PTZ) and anxiolytic (DIA) drugs on inter-male aggression

In order to study the effects of pharmacological manipulation of anxiety-related behavior on aggression (Neumann et al., 2010), LAB rats (*n* = 23) were injected with the anxiogenic drug PTZ (25 mg/kg, ip) (Cruz et al., 1994), HAB rats (*n* = 24) with the anxiolytic drug DIA (2 mg/kg, ip) (Pellow et al., 1985) and NAB rats (*n* = 23) with both PTZ or DIA; respective controls received VEH (Ringer's solution, 1 ml/kg; ip). Our pilot experiments (data not shown) showed that the low anxiety-related behavior of male LAB rats cannot be further reduced by DIA, thus, they were only injected with PTZ. Similarly, the high anxiety level of HAB rats cannot be further increased by PTZ; therefore, they were only treated with DIA. Thirty min after the injection, the experimental rats were tested for aggressive behavior in the RI test. Additional groups of LAB (*n* = 12) and HAB (*n* = 16) males were treated with PTZ or DIA, respectively, and their anxiety-related behavior was tested on the EPM. The anxiolytic and anxiogenic effects of DIA and PTZ, respectively, on the EPM in non-selected Wistar rats are already very well established (Pellow et al., 1985; Cruz et al., 1994).

### Histology

To verify the infusion sites, rats were killed by an overdose of anesthetics after the end of the behavioral tests. Icv brains were infused via the guide cannula with ink (5 μl), instantly cut coronally, and checked for staining of the ventricle. Locally infused brains were frozen in pre-chilled *n*-methylbutane on dry ice, and infusion sites were localized on 40-μm coronal cryostat sections stained with cresyl violet.

### Statistics

All statistical analyses were performed using the software package SPSS (version 19). Behavioral parameters of the RI test were analyzed using either Students *t*-test, when two treatments were compared (e.g., vehicle vs. NPS, or vehicle vs. PTZ) or a One-Way analysis of variance (ANOVA) followed by a *post-hoc* analysis using Bonferroni correction, if appropriate, in case three treatments groups were compared (e.g., Vehicle, PTZ, DIA). If variance equality was violated, adjusted *p*-values together with unadjusted degrees of freedom are presented with the *t*-values. Separate statistical analysis of treatment effects as described above has been performed on LAB, HAB, and NAB rats, as experiments on the 3 rat lines were performed at different times. However, the parameter overall aggressive behavior has been compared between all three lines using a Two-Way ANOVA (factors rat line × treatment). Social investigation in the social preference test was compared using a 2 × (2) mixed model ANOVA (drug treatment [between subject] × stimulus [within-subject]) followed by a *post-hoc* comparison using Bonferroni correction. EPM behavior was analyzed using Student's *t*-test. Data are presented as mean + standard error of the mean (s.e.m.). Statistical significance was set at *p* < 0.05.

## Results

### Experiment 1: effects of icv NPs on inter-male aggression in LAB, HAB and NAB males and effects on social preference in LAB and HAB rats

#### RI test

**Figure 1:**
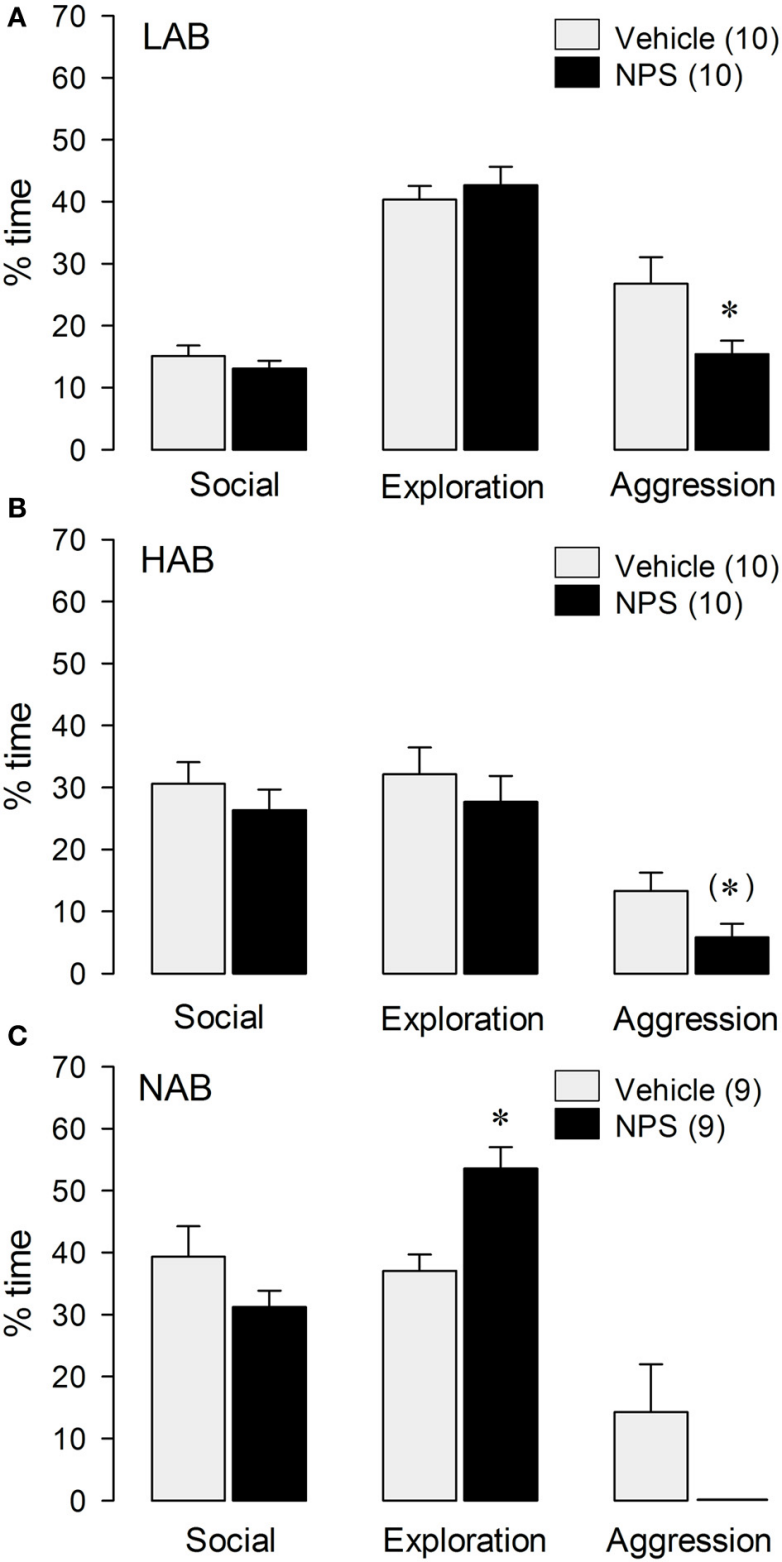
**Behavior of LAB (A), HAB (B), and NAB (C) rats during the RI test**. NPS (1 nmol) was applied icv 30 min before testing. Social investigation, cage exploration, and aggressive behavior are calculated as percentage of total time (10 min). Numbers in parentheses indicate group size. Date are means + s.e.m., ^*^p < 0.05 vs. vehicle, (^*^) 0.1 > *p* > 0.05. Student's *t*-test.

LAB rats treated with icv NPS (1 nmol) displayed less total aggressive behavior [*t*_(18)_ = 2.38; *p* < 0.05; Figure 1A] and spent more time immobile (VEH: 3.9% ± 0.9, NPS: 12% ± 3.0) [*t*_(18)_ = −2.62; *p* < 0.05] in the RI test compared with VEH-treated LABs. No significant differences between NPS- and VEH-treated LAB residents were found in social investigation, exploration or any other behavior investigated (Figure 1A). There were no significant treatment effects on distinct aggressive parameters.

In HAB rats, a trend toward reduced total aggressive behavior [*t*_(18)_ = 2.03; *p* = 0.057; Figure 1B], and an increase in immobility (VEH: 4.9% ± 1.4, NPS: 28% ± 9.0) [*t*_(18)_ = 2.59; *p* < 0.05; Table 1] were found after icv NPS-treatment compared with VEH. No significant differences between NPS- and VEH-treated rats were found with respect to social investigation and exploration or any other behaviors investigated (Figure 1B).

**Table 1:** **Anxiety-related behavior of LAB, HAB, and NAB rats on the elevated plus-maze (EPM) and home cage immobility during the resident- intruder (RI) test**.

**Paradigm**	**EPM**				**RI (home cage)**	
**Readout**	**Time open arm (%)**		**Entries closed arm**		**Immobility (%)**	
**Treatment**	**Veh**	**Drug**	**Veh**	**Drug**	**Veh**	**Drug**
LAB (NPS/NAc)	69.2 ± 3.3	71.6 ± 5.4	6.5 ± 0.7	5.6 ± 0.8	7.2 ± 2.3	5.5 ± 1.0
LAB (NPS/LH)	65.3 ± 3.7	71.5 ± 4.9	4.6 ± 0.7	6.7 ± 0.5^*^	4.6 ± 1.0	6.8 ± 2.3
LAB (PTZ/ip)	76.1 ± 4.2	44.3 ± 6.2^*^	6.3 ± 0.9	5.3 ± 0.3	3.5 ± 0.67	9.3 ± 2.3^*^
HAB (DIA/ip)	3.2 ± 2.2	16.6 ± 6.0^*^	1.7 ± 0.7	2.6 ± 0.6	3.0 ± 0.82	9.2 ± 2.5^*^

*Neuropeptide S (NPS, 0.2 nmol) was applied bilaterally in the nucleus accumbens (NAc) or the lateral hypothalamus (LH) 30 min before testing. Pentylenetetrazol (PTZ, 25 mg/kg) and Diazepam (DIA, 2 mg/kg) were injected ip, 30 min before testing. Date are means ± s.e.m.*,

* p < 0.05 vs. vehicle. Student's t-test.

NPS-treated NAB rats spent more time with cage exploration [*t*_(16)_ = −3.80; *p* < 0.01 vs. VEH], whereas social investigation, aggressive behavior (Figure 1C) and immobility (data not shown) did not significantly differ between the NAB treatment groups.

However, overall statistics of aggressive behavior on all three strains, reveals a significant reduction of aggression between NPS- and VEH-treated rats [*F*_(1, 52)_ = 12.1; *p* < 0.005], indicating a general anti-aggressive effect of NPS (Figure 1). Overall statistics did not reveal an interaction effect [strain × treatment; *F*_(2, 52)_ = 55.4; *p* = 0.685].

#### Social preference

**Figure 2:**
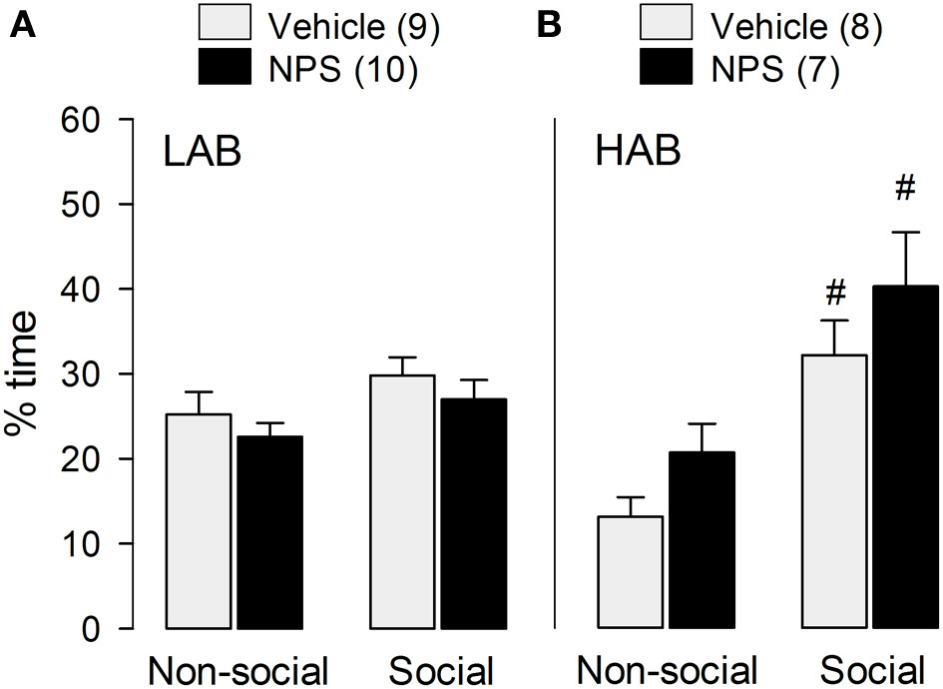
**Behavior of LAB (A) and HAB (B) rats in the social preference test**. NPS (1 nmol) or vehicle were applied icv 30 min before testing. Social preference was reflected by the time the experimental rats spent sniffing the non-social (empty cage) and the social stimulus, respectively. NPS (1 nmol) was applied icv 30 min before testing. Time of non-social and social investigation are calculated as percentage of total time (4 min). Numbers in parentheses indicate group size. Data are means + s.e.m., #*p* < 0.05 vs. non-social stimulus; 2 × (2) mixed model ANOVA (drug treatment [between subject] × stimulus [within-subject]) followed by a *post-hoc* test (Bonferroni).

In confirmation of previous results found in male NAB rats (Lukas and Neumann, 2012), icv NPS did not alter social preference behavior in LAB [*F*_(1, 17)_ = 0.003; *p* = 0.96; Figure 2A] and HAB [*F*_(1, 13)_ = 0.004; *p* = 0.95; Figure 2B] rats as indicated by a similar percentage of time exploring the social stimulus compared with VEH-treated rats.

Whereas both VEH- (*p* < 0.01) as well as NPS-treated (*p* < 0.05) HAB males showed a preference for the social stimulus compared to the non-social stimulus (Figure 2B), in contrast, social preference behavior was missing in LAB males (Figure 2A).

### Experiment 2: no effects of local infusions of NPS into the NAc and LH on inter-male aggression and anxiety-related behavior in LAB rats

#### RI test

Bilateral infusion of NPS into the NAc (VEH: 21% ± 5.5; NPS [0.1 nmol]: 18% ± 2.3; NPS [0.2 nmol]: 15% ± 4.2) and the LH (Vehicle: 55% ± 6.8; NPS [0.2 nmol]: 60% ± 5.1) did not change total aggressive behavior.

#### EPM

Similarly, LAB rats bilaterally infused with NPS (0.2 nmol) either into the NAc or the LH did not differ from VEH-treated rats in the percentage of time spent on the open arms of the EPM (Table 1). Although there were no changes on home cage locomotion following NPS infusion into the LH in the RI test, NPS infusion into the LH significantly increased the number of entries in the closed arms [*t*_(20)_ = −2.64; *p* < 0.05 vs. VEH; Table 1].

### Experiment 3: effects of ip PTZ and DIA on inter-male aggression and anxiety-related behavior

#### RI test

**Figure 3:**
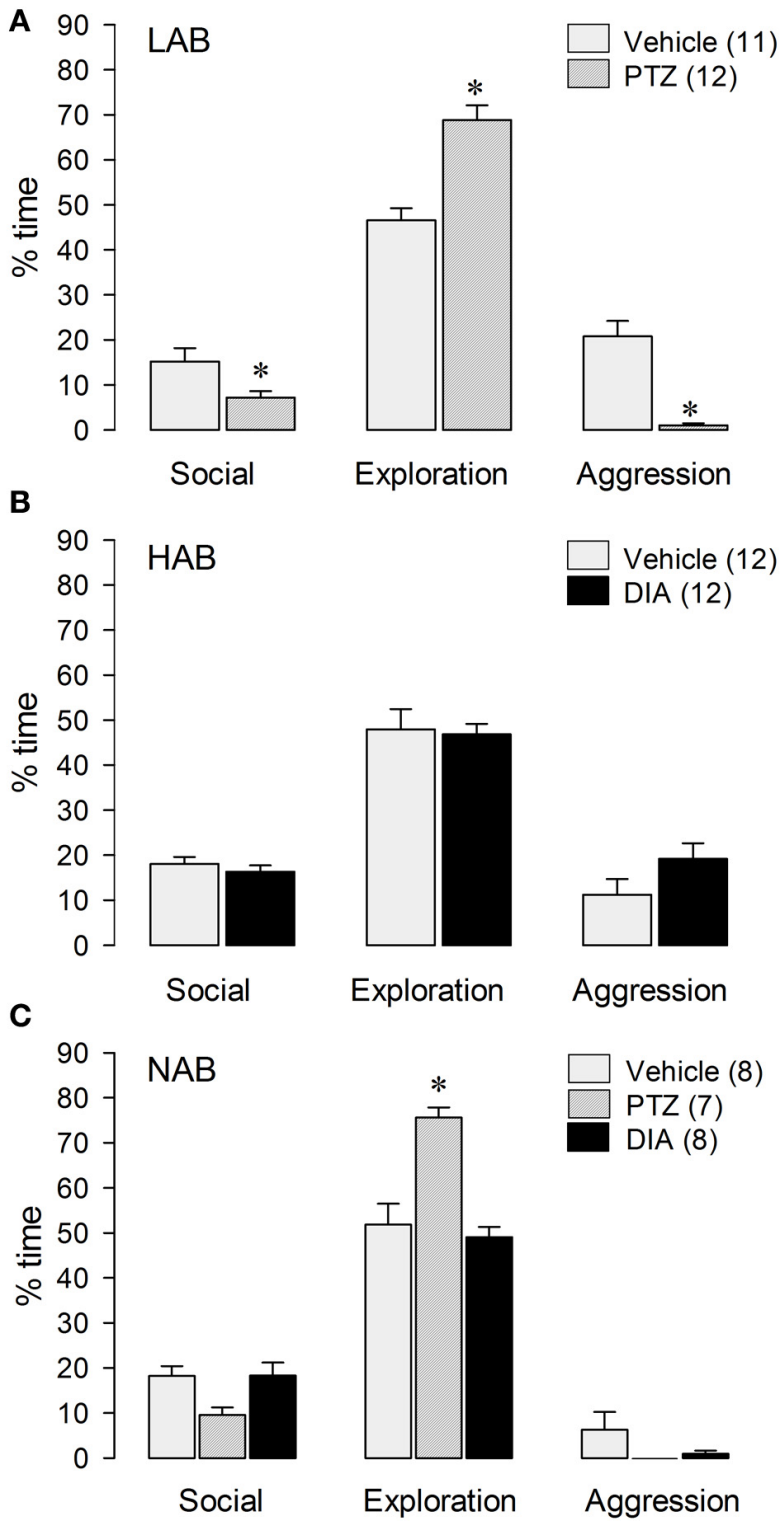
**Behavior of LAB (A), HAB (B), and NAB (C) rats during the RI test**. Pentylenetetrazol (PTZ, 25 mg/kg) and Diazepam (DIA, 2 mg/kg) were applied ip 30 min before testing. Social investigation, cage exploration, and aggressive behavior are calculated as percentage of total time (10 min). Numbers in parentheses indicate group size. Date are means + s.e.m., ^*^p < 0.05 vs. vehicle. One-Way-ANOVA followed by *post-hoc* test (Bonferroni) and student's *t*-test, respectively.

LAB residents treated with the anxiogenic drug PTZ displayed less inter-male aggression [*t*_(21)_ = 5.72; *p* < 0.001] and social investigation [*t*_(21)_ = 2.48; *p* < 0.05], but more exploration [*t*_(21)_ = −5.21; *p* < 0.001; Figure 3A] and immobility [*t*_(21)_ = −2.41; *p* = 0.05; Table 1] compared with VEH-treated males.

In contrast, HAB males treated with the anxiogenic drug DIA displayed more immobility [*t*_(22)_ = −2.37; *p* < 0.05 vs. VEH; Table 1], whereas aggression, social investigation and exploration remained unchanged after DIA-treatment (Figure 3B).

In NAB residents treated with either PTZ, DIA or vehicle (Figure 3C), there was no treatment effect on total aggression [*F*_(2, 20)_ = 2.02; *p* = 0.16] or immobility [*F*_(2, 20)_ = 0.90; *p* = 0.42]. In contrast, there was a significant treatment effect on social investigation [*F*_(2, 20)_ = 4.35; *p* < 0.05] and exploration [*F*_(2, 20)_ = 18.3; *p* < 0.001]. In detail, PTZ-treated NAB males spent more time with exploration (*p* < 0.001) than VEH- as well as DIA-treated NAB rats (Figure 3C).

#### EPM

The established anxiogenic and anxiolytic effects of PTZ and DIA (Pellow et al., 1985; Cruz et al., 1994) were confirmed in LAB and HAB rats, respectively. PTZ-treated male LAB rats spent less time on the open arms of the EPM [*t*_(10)_ = 4.25; *p* < 0.01 vs. VEH; Table 1] indicating an increase in anxiety-related behavior, whereas the number of open arm entries remained unchanged. In contrast, DIA-treated HAB rats showed increased time on the open arms [*t*_(14)_ = −2.31; *p* < 0.05 vs. VEH; Table 1] with an unchanged number of open arm entries.

## Discussion

In the present study, we describe for the first time that central NPS modulates social behavior in rats, as it reduces inter-male aggression regardless of the innate aggression level. However, attempts to localize the anti-aggressive effect of NPS in brain regions involved in reward and aggression, namely the NAc and the LH, failed suggesting that NPS may mediate these effects in other brain regions or indirectly via modulation of anxiety. In order to investigate the latter hypothesis, LAB, HAB and NAB rats were treated with either established anxiogenic drug PTZ or the anxiolytics DIA (Pellow et al., 1985; Cruz et al., 1994; Liebsch et al., 1998). Increasing anxiety in male LAB rats via PTZ resulted in a reduction of aggressive behavior accompanied by a reduction in social investigation, whereas reducing anxiety in HAB rats via DIA did not affect aggression.

Former studies have demonstrated potent properties of NPS to reduce anxiety- and fear-related behavior (Xu et al., 2004; Jüngling et al., 2008; Ionescu et al., 2012; Lukas and Neumann, 2012; Slattery et al., 2012; Dine et al., 2013), and to modulate learning and memory (Han et al., 2009; Okamura et al., 2011; Lukas and Neumann, 2012) when applied either icv or locally into the amygdala and ventral hippocampus in rats and mice. In contrast, social behaviors including social approach, social anxiety, and social memory were not altered by synthetic NPS, and the endogenous NPS system does not seem to contribute to the regulation of these aspects of social behavior (Lukas and Neumann, 2012). However, our findings provide the first evidence for the involvement of NPS in the regulation of inter-male aggression. The strongest anti-aggressive effect of icv NPS was found in LAB rats, which show the highest level of aggression. In contrast, the aggression-reducing effect of NPS was less pronounced in HAB and NAB males, probably due to their generally lower level of aggression in the RI test (Beiderbeck et al., 2012). Additionally, the aggression-reducing effect of NPS could be based on the reduction of rewarding effects of aggression, which were only found in LAB rats (Beiderbeck et al., 2012). Further, differences in the endogenous NPS system between LAB, HAB, and NAB rats have been demonstrated. In particular, increased NPSR expression in LAB compared to HAB males within the hypothalamic paraventricular nucleus (PVN) of rats and the medial amygdala of mice (Slattery et al., 2012) may begin to explain the differences observed in the present study.

Importantly, the NPS-induced decrease in aggression is not due to a general reduction in social motivation, as the time rats spend in non-aggressive social investigation during the RI test and social approach behavior in the social preference test was not altered by NPS in any of the groups. These results are in confirmation of our recent findings of NPS exerting anxiolytic (Slattery et al., 2012) and memory-enhancing (Lukas and Neumann, 2012) effects only in a non-social context without altering social preference behavior or social discrimination abilities.

Former studies have repeatedly shown increased locomotion and arousal after central NPS treatment, when testing was performed in unknown, stressful environments or in the early light phase (Xu et al., 2004; Smith et al., 2006; Rizzi et al., 2008). In contrast, we found a significant increase in immobility after icv NPS in both LAB and HAB rats during the RI test. This may be a consequence of the reduced time these rats spent with aggressive behavior. In our experiments, residents were tested in their home cage during the early dark phase, i.e., the active phase of rats, which may further explain the lack of arousal after NPS-treatment.

In an attempt to identify the brain region(s) responsible for the anti-aggressive effect of NPS we selected two regions, the NAc and LH, characterized by both the presence of NPSRs (Leonard and Ring, 2011) and by increased neuronal activity in response to the display of high aggression in LAB residents (Beiderbeck et al., 2012). In addition to the NAc and the LH, NPSRs were identified in several brain regions belonging to the reward system and in the hypothalamic part of the stress axis including the ventral tegmental area and the arcuate hypothalamic nucleus, as well as in other regions involved in the regulation of aggression like the anterior and dorsal hypothalamic area (Leonard and Ring, 2011). Furthermore, the NAc was selected as a target region as we have recently demonstrated that the high aggression level of LAB rats is mediated via the mesolimbic reward system, i.e., the dopamine system in the NAc (Beiderbeck et al., 2012). As NPS may directly act on brain regions known to be important for the regulation of aggression, we also chose the LH for local NPS infusions (Tulogdi et al., 2010). This was supported by the finding of Kallupi et al. (2010) demonstrating that icv infusion of NPS triggers neuronal activation in the LH in mice.

Although previously shown to exert local effects on anxiety in other brain regions (Jüngling et al., 2008; Ionescu et al., 2012; Slattery et al., 2012; Dine et al., 2013) bilateral infusion of NPS into either the NAc or LH prior to behavioral testing did not alter inter-male aggression in the RI test or anxiety-related behavior on the EPM. Although it cannot be excluded that higher doses of NPS may be effective, this suggests that these two regions are not mediating the anxiolytic and anti-aggressive effects of NPS. Thus, further studies are needed to localize the aggression-reducing effect of NPS. Potential target regions are the anterior hypothalamus and the arcuate nucleus, which are not only rich in NPSRs (Leonard and Ring, 2011), but also implicated in the regulation of aggression (Veening et al., 2005; Siever, 2008; Beiderbeck et al., 2012). Furthermore, the ventral tegmental area, which is also rich of NPSRs and represents the upstream element of the dopaminergic reward system, could be a target for future studies, but local neuronal activation did not increase during the display of high aggression in LAB rats (Beiderbeck et al., 2012). Additionally, further studies should investigate, whether infusion of NPS into regions involved in its anxiolytic effect, like the amygdala or the ventral hippocampus (Jüngling et al., 2008; Dine et al., 2013) also leads to a reduction of aggression in male rats. Another option for indirect regulation of aggression is that hypothalamic regions linked to the regulation of hypothalamus-pituitary-adrenal (HPA) axis activity, for example the PVN, may be of importance for the anti-aggressive effects of NPS. Alterations in the reactivity of the HPA axis to social stressors may also underlie behavioral changes in the RI test. However, both high and low HPA axis reactivity have been associated with high aggression levels (Haller and Kruk, 2006; Veenema et al., 2007; Neumann et al., 2010), resulting in the hypothesis of reactive vs. non-reactive aggression (Koolhaas et al., 1999). This opens the route for speculations that NPS reduces aggression via modulating the HPA axis and thereby the social stress response. However, HPA response after aggressive encounters is already increased in highly aggressive LAB rats (Veenema et al., 2007), and NPS is considered to result in a further activation of the HPA axis; at least under basal conditions (Smith et al., 2006).

Finally, we investigated whether the effect of NPS on aggression could be due to its robust anxiolytic properties. Thus, LAB and HAB males were treated with established anxiogenic (PTZ) and anxiolytic (DIA) substances prior to exposure to the RI test, respectively. PTZ-treatment of LAB rats indeed increased their low basal anxiety level, and reduced their high level of aggressive behavior in the RI test compared with vehicle-treated LAB males. Moreover, DIA-treatment of HAB rats resulted in reduced anxiety levels as seen after NPS treatment, but did not alter inter-male aggression. In NAB rats, neither PTZ nor DIA altered their low level of aggression in the RI test. In the last years, the potential link between aggression and anxiety has been discussed controversially (for review see Neumann et al., 2010). Such a link has been shown, for example, in different strains of mice (Cases et al., 1995; Oliveira-Dos-Santos et al., 2000; Nyberg et al., 2003), whereas other studies did not find such a link. For example, enkephalin knockout mice and North Carolina mice show both increased inter-male aggression and increased anxiety (Nehrenberg et al., 2009). In LAB and HAB rats, both individuals with low and high innate anxiety levels are more aggressive compared with NAB rats. However, LAB males display not only the highest level, but also abnormal forms of aggression (Beiderbeck et al., 2012). Additionally, mice bred for short- or long-attack latency do not differ in anxiety-related behavior (Hogg et al., 2000). Also, oxytocin knockout mice characterized by low levels of anxiety do not consistently differ in aggression compared with wild-type mice (Winslow et al., 2000). Thus, it has been suggested that aggression and anxiety can be independently regulated. In support, highly aggressive North Carolina mice show a reduction in both aggression and anxiety when treated with DIA (Nehrenberg et al., 2009), whereas the HAB rats in the present study did not show a decrease in aggression after DIA-treatment. Also, increasing anxiety-related behavior by PTZ, and infusion of the anxiolytic NPS reduced aggression in LAB rats. Taken together, there are multiple studies rather suggesting lack of a direct link between and independent regulation of aggression and anxiety. Therefore, the anti-aggressive properties of NPS are not likely to be caused by its strong anxiolytic effect.

In conclusion, central infusion of NPS is effective to reduce inter-male aggression in rats, an effect which was not accompanied by a reduction of social interaction or social preference. Our experiments further suggest that the anti-aggressive effect of NPS is independent of its anxiolytic action, as alterations in anxiety may or may not result in alterations in aggression. Thus, our study is the first to identify the regulatory capacity of NPS to modify social behavior.

## Author contributions

Daniela I. Beiderbeck, Michael Lukas, and Inga D. Neumann designed the study. Daniela I. Beiderbeck and Michael Lukas performed experiments, analyzed data, and wrote the manuscript. Inga D. Neumann critically revised the manuscript for important intellectual content. All authors finally approved this version of the manuscript to be published and agreed to be accountable for all aspects of the work in ensuring that questions related to the accuracy or integrity of any part of the work are appropriately investigated and resolved.

### Conflict of interest statement

The authors declare that the research was conducted in the absence of any commercial or financial relationships that could be construed as a potential conflict of interest.
